# Towards functionally individualised designed footwear recommendation for overuse injury prevention: a scoping review

**DOI:** 10.1186/s13102-023-00760-x

**Published:** 2023-11-11

**Authors:** Patrick Mai, Leon Robertz, Johanna Robbin, Kevin Bill, Gillian Weir, Markus Kurz, Matthieu B. Trudeau, Karsten Hollander, Joseph Hamill, Steffen Willwacher

**Affiliations:** 1 Institute of Biomechanics and Orthopaedics, German Sport University Cologne, Am Sportpark Müngersdorf 6, 50933 Cologne, Germany; 2 Institute for Advanced Biomechanics and Motion Studies, Offenburg University, Offenburg, Germany; 3 Department of Physical Performance, Norwegian School of Sports Sciences, Oslo, Norway; 4 Biomechanics Laboratory, University of Massachusetts Amherst, Amherst, MA USA; 5 Sports Tech Research Centre, Mid Sweden University, Östersund, Sweden; 6 Google, Mountain View, CA USA; 7 Institute of Interdisciplinary Exercise Science and Sports Medicine, MSH Medical School Hamburg, Hamburg, Germany

**Keywords:** Running shoe, Customised, Additive manufacturing, Injury risk factor

## Abstract

Injury prevention is essential in running due to the risk of overuse injury development. Tailoring running shoes to individual needs may be a promising strategy to reduce this risk. Novel manufacturing processes allow the production of individualised running shoes that incorporate features that meet individual biomechanical and experiential needs. However, specific ways to individualise footwear to reduce injury risk are poorly understood. Therefore, this scoping review provides an overview of (1) footwear design features that have the potential for individualisation; and (2) the literature on the differential responses to footwear design features between selected groups of individuals. These purposes focus exclusively on reducing the risk of overuse injuries. We included studies in the English language on adults that analysed: (1) potential interaction effects between footwear design features and subgroups of runners or covariates (e.g., age, sex) for running-related biomechanical risk factors or injury incidences; (2) footwear comfort perception for a systematically modified footwear design feature. Most of the included articles (*n* = 107) analysed male runners. Female runners may be more susceptible to footwear-induced changes and overuse injury development; future research should target more heterogonous sampling. Several footwear design features (e.g., midsole characteristics, upper, outsole profile) show potential for individualisation. However, the literature addressing individualised footwear solutions and the potential to reduce biomechanical risk factors is limited. Future studies should leverage more extensive data collections considering relevant covariates and subgroups while systematically modifying isolated footwear design features to inform footwear individualisation.

**Supplementary Information:**

The online version contains supplementary material available at 10.1186/s13102-023-00760-x.

## Key points

Individualised footwear bears the potential to alter biomechanics and, subsequently, the individual injury risk profile. Since different footwear modifications modulate risk factors for specific overuse injuries diffently, individualised footwear solutions must be designed for specific overuse injuries by targeting injury-specific biomechanical risk factors. The influence of various footwear modifications on biomechanical risk factors concerning environmental constraints, athlete anthropometry, and experience level has been documented only to a limited extent and requires further research. In addition to minimising biomechanical risk factors, the comfort and fit of the shoe should be considered to design an individually optimal shoe.

**Supplementary Information:**

The online version contains supplementary material available at 10.1186/s13102-023-00760-x.

## Introduction

**Fig. 1:**
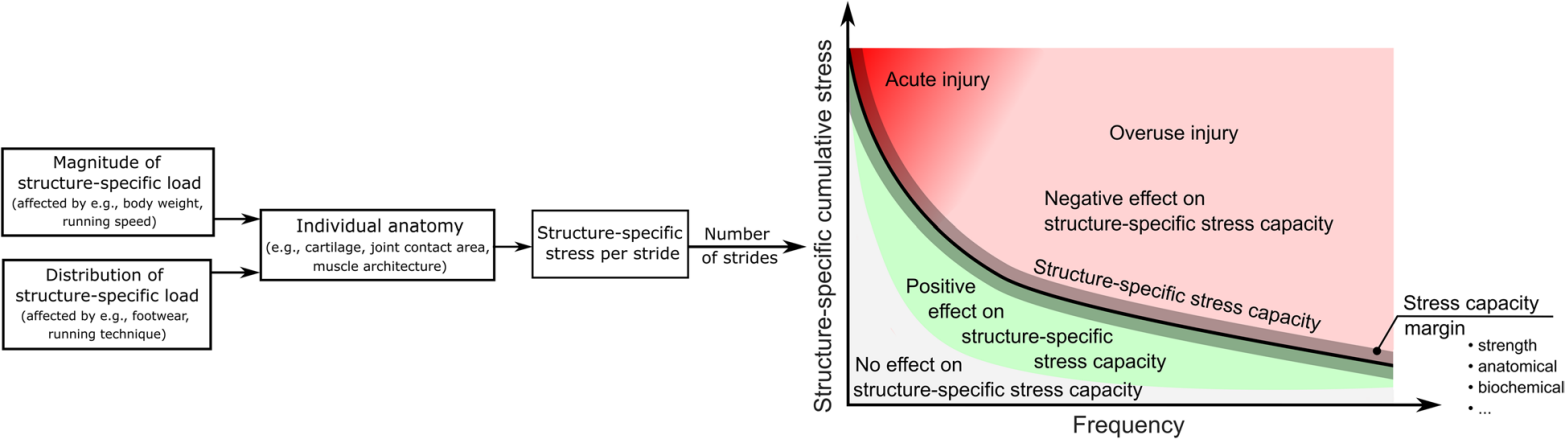
Interaction of risk factors that influence the workload of biological tissues. Schematic overview of an individual injury risk profile. Accumulated risk for a specific overuse injury is the sum of non-modifiable and modifiable risk factors

Running is one of the most popular global sports activities, likely because of its positive impact on physical and mental health and the simplicity of the equipment needed [1–3]. According to a 2020 data report from Strava, a social network for athletes, more than 3 billion kilometers were covered by this running community in 2020 alone [4]. However, with injury prevalences up to 79%, running is also associated with a high incidence of running-related overuse injuries (RRI) [5]. The onset of RRI is multifactorial. Risk factors are typically framed as intrinsic (e.g., genes, sex, age) and extrinsic (e.g., biomechanical, training-related, environmental) [6]. Other frameworks center around the causal biological mechanism to understand the multifactorial etiology of RRI [7]. Mechanically, the onset of RRI is a result of continuously exceeding the structure-specific stress capacity without sufficient resting periods for tissue remodeling (Fig. 1). Directly determining the individual structure-specific stress characteristics (e.g., amplitude, duration, frequency, distribution) and the underlying neuromuscular control strategies remain challenging [8, 9]. In the complex puzzle of RRI, biomechanical risk factors (BRFs) are surrogate variables that link running biomechanics and injury risk [10]. Mechanically, footwear is believed to reduce BRFs by altering the distribution of structure-specific stress applied per stride (short term), reducing the cumulative stress within and across multiple running sessions. As a result, running footwear can affect the positive adaptation of biological tissue (long term), allowing the tissue greater capacity to tolerate more stress [6].

Since the introduction of the first commercial running shoe in the early twentieth century, the footwear industry has seen significant evolution, notably by the 1970s development of cushioned midsoles aiming to attenuate the vertical impact force [11, 12]. However, thicker, more cushioned midsoles resulted in an inherently less stable base of support [11]. In the early 1990s, footwear manufacturers introduced motion control technologies to support biomechanical foot stability, a concept still largely governed today by foot guidance and shock attenuation principles. [11–13]. While running shoes are commercially available in a multitude of different designs and functions nowadays, limited research focuses on the specificity of footwear relative to an individual's anatomy and biomechanics and injury rates remain high [14]. Currently, there is limited evidence that running footwear with stability and cushioning elements could reduce RRI rates [15]. However, drastic footwear modifications such as oversimplified designs (e.g., minimal shoes) or over-designed modifications, combined with insufficient habituation time, can result in the onset of RRI [16–18]. New approaches, e.g., the comfort filter, habitual motion path theory, and the preferred movement path theory, linking RRIs to uncomfortable perceived shoes, are becoming increasingly established in the running community and may serve as an opportunity to reduce injuries in the future [12, 19, 20]. These new concepts aim to enhance the subject-specific response to footwear.

**Fig. 2:**
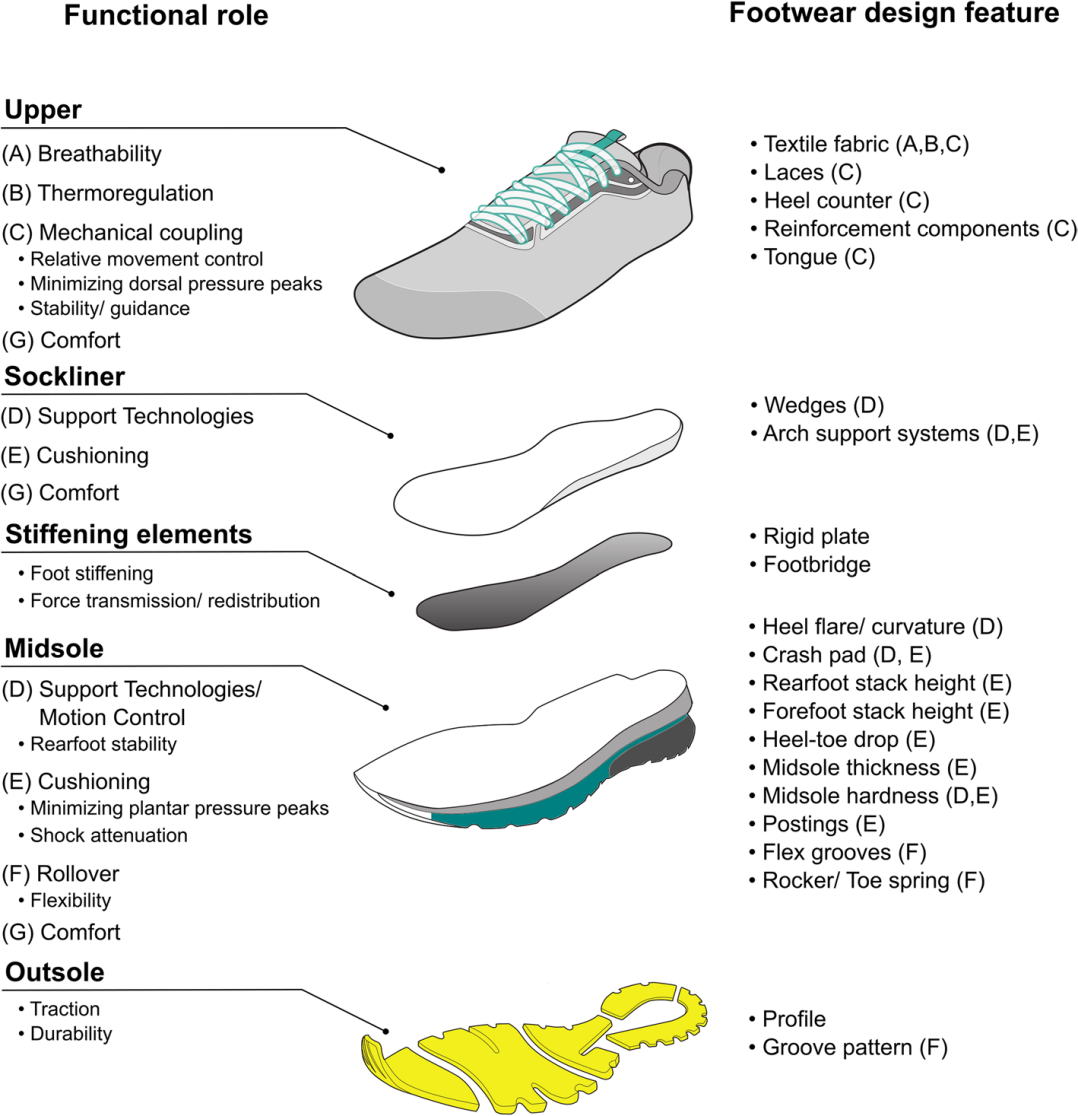
An assembly drawing of the various construction elements of common running footwear with their functional roles (left-hand side). Categorisation of different footwear design features with the fulfilled function (right-hand side). Letters in brackets link the functional role of footwear to its modifiable footwear design feature(s) (e.g., breathability can be changed by manipulating the textile fabric)

Understanding the individual response to footwear is critical since the number of footwear design features (FDF) has increased substantially (Fig. 2). The individualisation of footwear is further motivated by the observation that today's running community is as diverse as ever in human history [12]. Runners differ in their experience levels, running behaviours, strength capacities, and anthropometrics. In the past, the individualisation of footwear was time- and cost-intensive and, thus, mostly limited to elite athletes. Today, rapid additive manufacturing processes (e.g., 3D printing) enable individualised midsoles and fabrics for a broader audience [21, 22]. These manufacturing technologies might further allow footwear functions to match runners' individual needs, thus potentially reducing the risk of RRI. However, it is unclear which FDF bear the highest potential for individualisation. Further, it is not well understood which characteristics (e.g., age, sex, training environment) of runners determine the details of the individualisation process. Although shoe orthopedists have supplied runners with customised footwear for decades, the modifications of these features are mainly based on experience due to the limited scientific evidence available. Therefore, the purpose of this scoping review was to provide: (1) an overview of footwear design features that might have a potential for individualisation; and (2) a systematic overview of the literature on the differential response to footwear design features between selected groups of individuals. In particular, the potential of running shoe individualisation to reduce the magnitudes of injury-specific BRFs will be evaluated.

## Methods

**Fig. 3:**
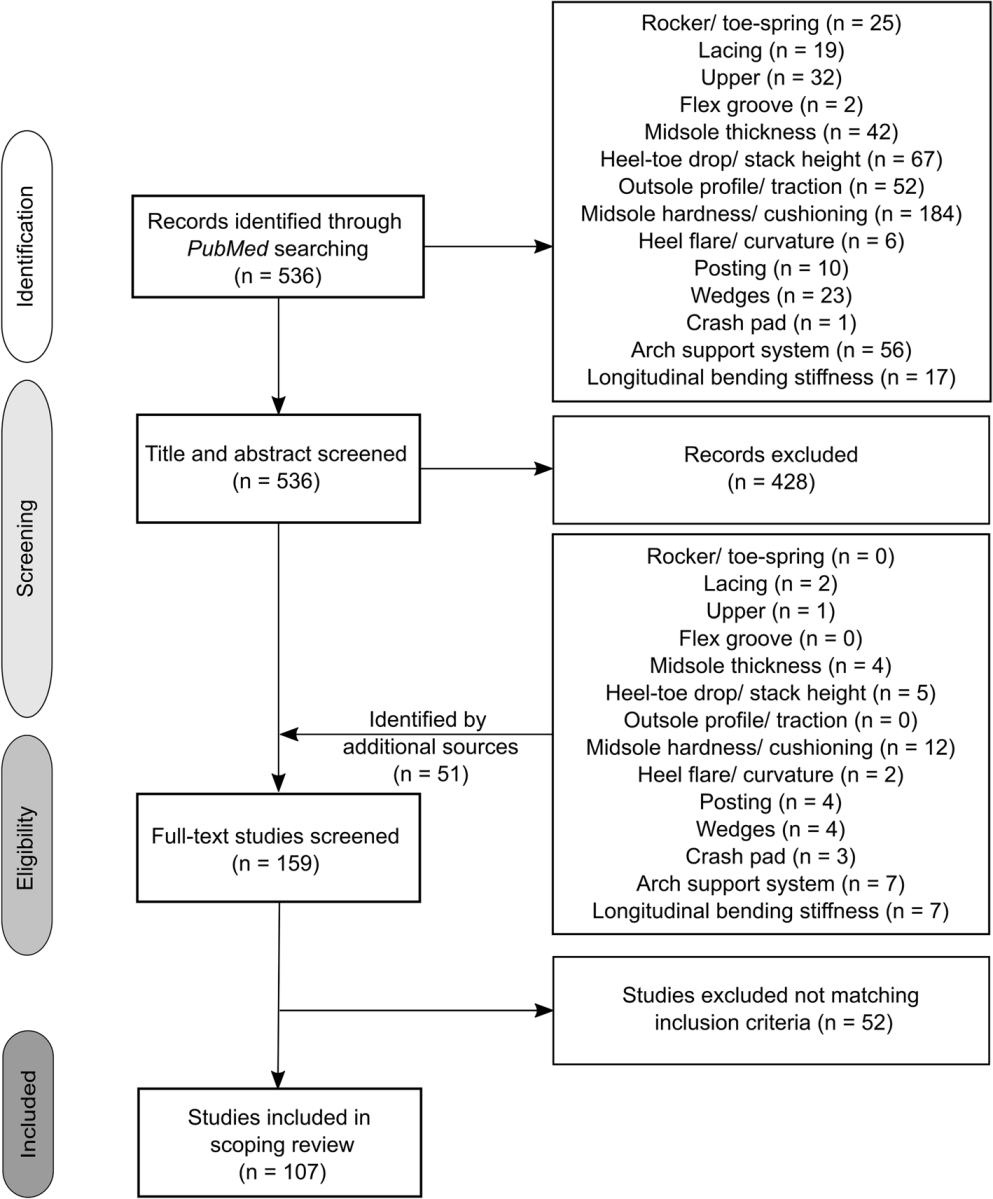
The flow chart of the search and screening procedure

The review team drafted and revised the scoping review protocol [23] using the PRISMA checklist ( Supplementary Information (SI1). For the scoping review, we included studies in the English language on adults that analysed: (1) the interaction effects between footwear design features and subgroups of runners or covariates (e.g., age, sex) on BRFs; (2) BRFs without considering covariates; (3) injury incidences and (4) footwear perception for a systematically modified footwear design feature. Although studies that ignore covariates do not immediately inform different subgroups of runners about a running shoe lowering injury risk, they may assist in identifying a systematic shoe response and thus help design FDF graduations. We selected the BRFs for the most common overuse injuries (Table 1) from a recently published systematic review [10]. The most common overuse injuries in distance running include Achilles tendinopathy (AT), tibial stress fractures (TSF), plantar fasciitis (PF), iliotibial band syndrome (ITBS), patella femoral pain syndrome (PFPS), medial tibial stress syndrome (MTSS) [5]. A systematic literature search was performed on the PubMed database (Fig. 3 between June 2021 and April 2022). We used FDF-specific search strings (see SI2 for details). Each search string contained items to identify articles that analysed the effect of footwear while running (runn* OR jogg*). The second item of the search string described a specific FDF or the function of a feature (e.g., midsole AND hardness OR cushion*). To address publications on footwear research only, we applied an additional combination of search string items (footwear OR shoe OR shod). Five authors screened titles and abstracts to reject irrelevant articles. Subsequently, two authors read each relevant article's full text and discussed it to assess their eligibility. During the full-text screening, relevant articles were additionally sourced through the reference lists and a co-citation method using the bibliographic coupling concept (www.connectedpapers.com). Further, data on study characteristics were extracted including publication details (author and year), population details (sample size), data collection methods, running speeds, covariates considered (if applicable), and biomechanical outcome variables, as well as a detailed description of the footwear studied.

**Table 1:** Summary of the running-related biomechanical risk factors (BRFs) and their level of evidence for the most common overuse injuries [10]. Arrows pointing upwards (downwards) indicate an increase (decrease) in the magnitude of the respective BRF associated with a specific overuse injury. Biomechanical risk factors in bold were identified in prospective studies; at least two independent retrospective studies identified unbolded BRFs. The supplementary information provides a detailed description of the BRFs (SI4)

	*Level of Evidence*	Achilles tendinopathy	Tibial stress fractures	Plantar fasciitis	Iliotibial band syndrome	Patello femoral pain syndrome	Medial tibial stress syndrome
Biomechanical related risk factor	*Moderate*			↑ Vertical instantaenous loading rate		↓ Ground reaction force braking impulse	**↑ Time spent in rearfoot eversion**
				↑ Vertical averange loading rate		↑ Ground contact time	**↑ Contralateral pelvic drop**
	*Limited*	**↓ Anterior–posterior displacement of the center of pressure**	↑ Peak rearfoot eversion angle			**↑ Knee abduction angular impulse**	
		**↑ Vertical forces at the lateral foot**	↑ Peak hip adduction angle			**↓ Time to peak force at medial and lateral heel**	
		**↓ Time to peak force at the medial heel**	↑ Peak free moment amplitude			**↑ Peak vertical force at lateral heel, metatarsal 2 and 3**	
		↑ Rearfoot inversion angle at initial contact					
	*Very Limited*				**↑ Iliotibial band strain**	**↑ Averange hip abduction moment**	
					**↑ Iliotibial band strain rate**		
					**↑ Peak femur external rotation angle**		
	*Inconsistent*	↑ Rearfoot eversion range of motion			**↑ Peak knee internal rotation angle**	**↑ Peak hip adduction angle**	**↑ Peak rearfoot eversion angle**
					↑ Peak knee adduction angle	**↑ Peak hip abduction moment**	
					↑ Knee flexion angle at initial contact	↑ Peak hip internal rotation angle	
						↑ Contralateral pelvic drop	
	*Conflicting*				**↑↓ Peak hip adduction angle**

## Results

### Overview

**Fig. 4:**
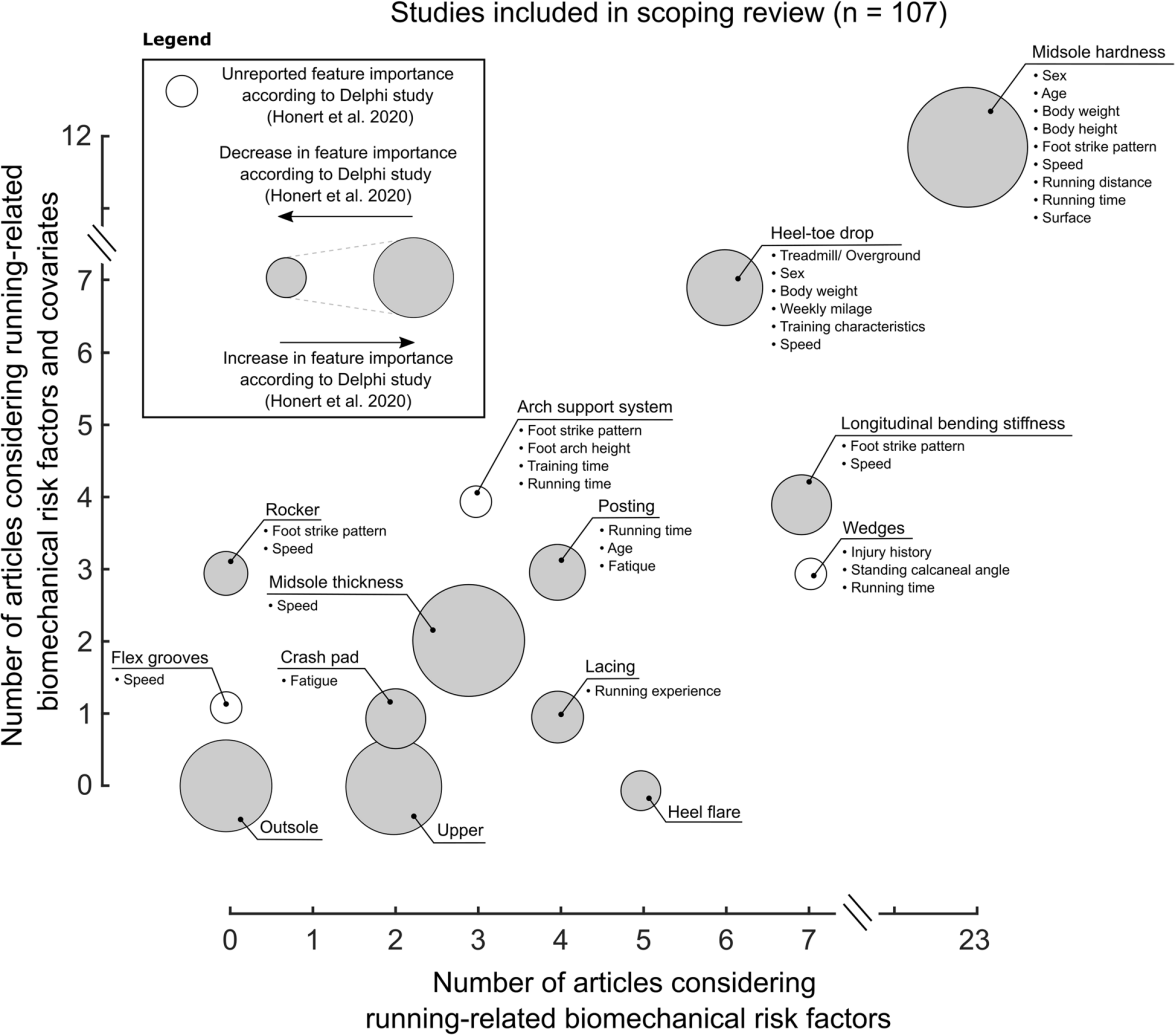
A Scatter plot of the included articles. Articles for each footwear design feature are separated by the number of articles considering covariates (y-axis) and running-related biomechanical risk factors (x-axis). If applicable, covariates for each footwear design feature are reported. According to a recent Delphi study, scatters are scaled to their importance [25]. Larger diameters represent a higher level of importance, and smaller diameters a lower level of importance. White scatters were not reported in the Delphi study and are not scaled

**Fig. 5:**
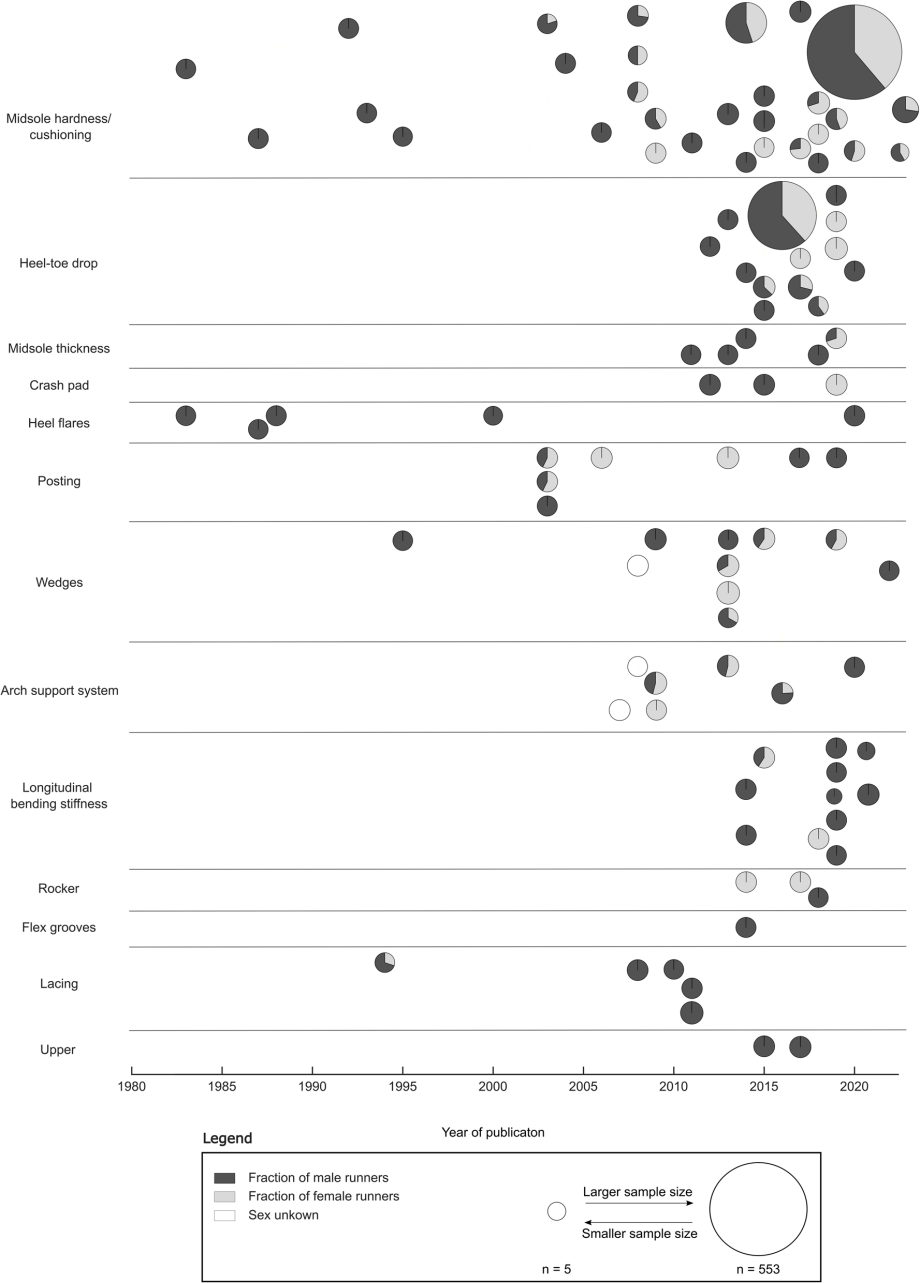
The publication timeline of the included articles, separated by the different footwear design features. Each pie chart represents one study with the fraction of male (dark-grey) and female (light-grey) runners. Pie charts are scaled to the number of runners included in the study. Larger diameters indicate larger sample sizes, and smaller diameters indicate smaller sample sizes

Running shoes are often characterised based on their cushioning and motion control functionality. Consequently, we have categorised the literature review results into these sections. We discuss additional FDF that did not fit into the first two sections in a subsequent part, followed by an upper construction segment. In each chapter, we introduce a brief description of the FDF. Next, we present the results of studies, taking covariates into account and analysing BRFs. We further discuss studies that investigated BRFs without considering covariates. Finally, we place our findings in the context of the FDF's potential to minimise the development of running-related overuse injuries (RRI). We identified 107 articles that met our inclusion criteria (Fig. 4, Supplementary 3 Table 1-12). Most of these articles were published at the start of the twenty-first century and primarily featured data from male runners (Fig. 5). We acknowledge a data gap in running footwear research, which aligns with the female data gap in sport and exercise science [24].

### Cushioning systems

Cushioned midsoles were one of the first FDF introduced to modern running shoes. They were developed to provide a protective layer, attenuate the shock caused by the collision of the foot with the ground, and reduce local plantar pressure peaks [26]. The cushioning characteristics are modified in the midsole through material and geometry changes.

#### Midsole compression stiffness and hardness

Midsole compression stiffness, also known as hardness, is a fundamental material property that measures the deformation caused by an area load. In the past, midsoles were constructed with uniformly distributed compression stiffness. However, they can now be tailored to individually cushioned midsoles with varying properties at different locations due to the viscoelastic properties of the material [27].

Twelve of thirty-five articles identified through our literature search considered covariates when analysing the response to differently cushioned midsoles (Fig. 4, Supplementary Table 1). Malisoux et al. considered the runner's body mass as a covariate [28]. Athletes reported fewer injuries when running in softer midsoles, and lighter runners in hard shoes showed a greater risk of developing an RRI than heavier runners. Three articles investigated the biomechanical response of midsoles with varying hardness during different running speeds. Nigg et al. found that the vertical GRF loading rate increases with speed independent of the cushioning variations, while another study showed unchanged GRF loading rates with footwear of varying cushioning at different speeds, and yet another study showed lower GRF loading rates in harder midsoles with no dependence on running speed [29–31]. Running distance or running duration has been considered by five studies [32–36]. None of the studies found significant footwear-by-time/distance interaction effects on vertical GRF loading rates, ground contact times, peak rearfoot eversion angles, and knee flexion angle at initial contact. One article considered the runner's foot strike pattern as a covariate [37]. Rearfoot strikers reduced the vertical GRF loading rate in a neutrally cushioned shoe, and mid- and forefoot strikers reduced the vertical GRF loading rate in a minimal shoe [37]. We identified one study considering the stiffness of the running surface as a covariate [38]. However, no main and interaction effects were observed in ground contact time and knee flexion angle at touchdown. Another study analysed the effect of surface inclination and midsole cushioning [39]. The authors showed that vertical GRF loading rates are equal when running on different surfaces with either a neutral or a cushioned running shoe. Although studies have examined a variety of covariates, there is much conjecture in the literature regarding their influence on biomechanical measures related to RRI, and no conclusive evidence to suggest that any one covariate is more important than another.

When considering the effects of midsole hardness on BRFs without considering covariates, five studies found reduced peak rearfoot eversion in harder midsoles than in softer midsoles [40–44]. However, four studies found unchanged peak rearfoot eversion angles when running in soft and hard midsoles [34, 45–47]. Four studies reported that different midsole hardness could not systematically affect the rearfoot eversion range of motion [40, 44, 45, 48]. In contrast, one study found a reduction in the rearfoot eversion range of motion in hard midsoles [47], and another study found that the range of motion of the rearfoot was lower when runners were running in softer midsoles [49]. Conflicting findings were also observed for the rearfoot inversion angle at initial ground contact. One study found a reduction in rearfoot inversion when running in soft midsoles [40], and others found reduced inversion angles when running in hard midsoles [40, 48]. Conflicting findings have also been reported for the vertical GRF loading rate. Some studies found an increased vertical GRF loading rate in more cushioned than less cushioned shoes [29, 34]. Other studies found no effects of cushioning [46, 50–52], while others found decreased vertical GRF loading rate in cushioned shoes [41]. Only a few studies were identified addressing the effects of different cushioning characteristics on BRFs at more proximal joints. One article's qualitative data showed that the knee abduction angle during the stance phase was reduced when running in softer than harder midsoles [53]. In contrast, another study found lower peak knee abduction angles when the midsole was manufactured with harder material [47]. A study by Malisoux and colleagues found that both soft and hard midsoles did not change peak hip abduction angles and moments and peak hip internal rotation angles [45]. When considering ground contact time as BRF for PFPS, most studies found no effect of midsole cushioning [29, 36, 38, 45, 49, 51, 54–56]. Overall, studies analyzing BRFs without considering covariates, resulted in inconsistent and conflicting findings. Interestingly, the footwear comfort perception reported by participants tends to be higher in regions where softer material is allocated than in those with harder materials [42, 49, 57, 58].

In summary, the current literature suggests that the midsole hardness can potentially reduce the overall injury risk when adjusted to the runner's body mass. Reduction in vertical GRF loading rates and subsequent minimizing PF injury risk could be achieved by individualising midsole cushioning to the runner's foot strike pattern. Specifically, rearfoot strikers might benefit from cushioned shoes, while fore- and midfoot strikers could find minimal shoes advantageous. The lower vertical GRF loading rates observed in neutral shoes compared to cushioned shoes when running downhill suggest that customised midsole cushioning tailored to a runner's training terrain could benefit runners with a PF history. Based on the limited literature, surface stiffness, running distance, and fatigue might be less important when individualising midsole hardness. Harder midsoles can reduce BRFs associated with MTSS, TSF, AT (rearfoot eversion movement), and ITBS (ground contact times). Indications that different shoe cushioning may alter vertical GRF loading rates are contradictory, and BRFs at more proximal joints have not been well studied.

#### Midsole geometry

Running footwear is often designed with a height gradient from the heel to the forefoot. Running shoes are defined by their heel and forefoot heights, with the difference between the two known as the heel-toe drop. Unlike neutral or motion-control shoes, minimal footwear is typically designed with a lower heel-toe drop. An increase in footwear minimalism generally shifts the foot strike pattern of rearfoot strikers towards a mid- or forefoot strike pattern, and it is further assumed to reduce impact loading parameters [59, 60].

We identified eighteen articles investigating the effects of geometrical midsole modifications matching our inclusion criteria (Fig. 4, Supplementary Table 2). Out of the eighteen articles, nine accounted for a covariate. The runner's experience was considered in one article [61]. During a six-month follow-up, it was shown that occasional runners (< 6 months running experience) had reduced injury rates, and recreational runners (≥ 6 months running experience) had increased injury rates when running in footwear with lower heel-toe drop. A subset of this data demonstrated that midsoles with different heel-toe drops were not able to reduce peak rearfoot eversion angle and ground contact time [62]. However, runners who trained for six months in footwear with higher heel-toe drops increased the peak knee abduction angle. On the contrary, runners who trained for six months in footwear with lower heel-toe drops reduced the peak knee abduction angle. Running surface as a covariate was considered by one study [63]. The researchers found smaller knee flexion angles for larger heel-toe drops when running on a treadmill. However, when running overground, the knee flexion angle was not changed when running in shoes with different heel-toe drops. The authors found that increasing the heel-toe drop led to lower vertical GRF loading rates overground, but decreasing the heel-toe drop reduced vertical GRF loading rates during treadmill running. Different running speeds as a covariate were considered by four articles [64–67]. One study found no changes in the knee flexion angle at initial contact when running at different speeds in midsoles with different heel-toe drop designs [64]. Another study showed that while ground contact time decreased with increasing speed, increasing the heel-toe drop resulted in increased contact time [65]. Other researchers also showed similar results when systematically altering running speed and heel-toe drop [66]. Running speed did not influence the effects of heel-toe drop modifications on vertical GRF loading rates or time spent in rearfoot eversion [67]. The interaction effects of running time and geometrical midsole modifications were investigated in two studies using the same data set [68, 69]. However, neither of the studies reported interaction effects on included BRFs (rearfoot movement, contact time, and knee flexion angle at initial ground contact). Nevertheless, both studies reported longer ground contact times, lower rearfoot eversion range of motion, and greater knee flexion angles at initial contact in thicker than thinner midsoles.

Concerning the general effects of midsole geometries on BRFs without considering covariates, most of the included studies have addressed the effect of midsole geometry on GRF parameters. An increase in heel-toe drop has been reported to reduce vertical GRF loading rates [70–73]. Diverse results have been reported for midsole thickness, for which one study found lower vertical GRF loading rates in thicker than thinner midsoles [74], whereas another study could not identify any differences [75]. Three studies showed that geometrical changes at the midsole do not affect rearfoot inversion at touchdown [68–70]. Three articles showed that the knee flexion angle at touchdown remains unchanged independent of geometrical midsole configurations [72, 75, 76]. Only one study collected comfort perception data from fifteen male runners [77]. However, no difference in comfort was observed when the heel-toe drop was systematically altered.

Summarising the results, individualisation of heel-toe drop based on runner experience may reduce the risk of RRI. Although the underlying biomechanical mechanism remains unknown, a gradual transition from shoes with different heel-to-toe drops may allow adequate adaptation of the biological tissues. Running surfaces can affect the response to heel-toe drop alterations by influencing vertical GRF loading rates and knee flexion angles. Runners with a history of PF training on treadmills may benefit from shoes with a lower heel-toe drop, while those with a history of ITBS may benefit from a higher drop. During fatigue, geometric midsole modifications may not affect rearfoot eversion movement or ground contact times. Thinner midsoles with a lower heel-toe drop may reduce ground contact times, peak rearfoot eversion angle and rearfoot eversion duration. Hence, these modifications might be recommended for runners with a risk or a history of PFPS, TSF, or MTSS. Moreover, thicker midsoles with a higher heel-toe drop might shift BRFs related to AT and PF (rearfoot eversion range of motion and vertical GRF loading rate) to potentially less critical BRF magnitudes.

### Motion control features

Motion control, also called stability, in footwear refers to how the shoe limits pronation (calcaneal eversion) or supination (calcaneal inversion) during the support phase. Much research has been devoted to FDF that purports to control pronation or eversion motion, motivated by the retrospective observations that increased pronation angle is associated with RRI [10, 78–80]. Over the initial period of footwear research, various midsole technologies were designed to increase rearfoot stability, including altering the midsole hardness, location of material inserts, flares, arch support systems, and postings. One of the few identified studies utilized a randomized controlled trial with a six-month follow-up. The findings revealed that recreational runners with a motion control shoe developed fewer RRI than runners receiving a standard running shoe [15]. Interestingly, motion-control shoes' effectiveness in reducing RRI development was more pronounced for runners with pronated feet, indicating some potential for footwear individualisation.

#### Postings

Postings in athletic footwear incorporate elements with higher material densities in the medial rearfoot region and have been reported to limit rearfoot eversion [81]. Unlike wedges, postings are designed without gradual height differences [82].

Three of seven articles identified through our literature search considered covariates in their analysis (Fig. 4, Supplementary Table 3). The runner's age was considered by one article [83]. Medial posts effectively reduced the amount of rearfoot eversion in older compared to younger female runners, while vertical GRF loading rates, peak knee abduction moments, and peak knee internal rotation angles remained unchanged. When considering the runners' fatigue as a covariate, two articles found that rearfoot eversion movement (peak and range of motion) was lower when running in a medially posted than in a neutral running shoe when the runner's fatigue increased [84, 85].

When not considering covariates or subgroups of runners, medial postings can reduce peak rearfoot eversion angles and eversion range of motion [86, 87]. Peak knee internal rotation angles are reported to be reduced when running in footwear with medial postings [83, 88]. However, footwear with postings might increase peak hip abduction moments [89]. Diverse results were found for vertical GRF loading rates. One study found lower vertical GRF loading rates in midsoles without medial posts [87], and another found unchanged vertical GRF loading rates in shoes with and without postings [83]. Some runners have perceived the harder posting material without transitions as uncomfortable, potentially resulting in unwanted changes in their biomechanics [88].

In summary, older female runners with a history of TSF and MTSS might reduce rearfoot eversion in shoes with postings. However, medial posts do not seem to affect the risk of developing PF independent of the runners' age since changes in vertical GRF loading rates were not observable. Based on the limited literature, posted midsoles may help minimise BRFs (rearfoot eversion movement) associated with AT, MTSS, or TSF as the runners’ fatigue state increases. The limited literature suggests that individualised postings can help runners with a history of AT, MTSS, TSF, or ITBS to reduce biomechanical risk factors. Since postings might increase vertical GRF loading rates, caution needs to be taken by runners with a history of PF.

#### Wedges

Wedges are sloped orthotic inserts, typically with mediolateral elevation, designed to increase foot stability. Mediolateral elevation under different loading conditions can be achieved by incorporating materials with different mechanical properties at distinguished locations of the wedge [90].

Three out of the ten articles identified in the literature search included a covariate in their analysis (Fig. 4, Supplementary Table 4). One study considered running duration (0–30 min) as a covariate [91]. Independent of the running duration, medially wedged insoles produced lower knee abduction angular impulses than laterally wedged insoles. Another study considered different standing calcaneal angles and injury history as covariates [92]. However, wearing differently wedged insoles showed no effect on female runners' 3D knee and hip kinematics. Anterior knee pain as a covariate and the response to differently wedged insoles were considered by one article [93]. Independent of knee pain, running in medially wedged insoles reduced maximal rearfoot eversion and range of motion compared to running in footwear without wedges. None of the studies personalised the wedges to the runner's individual foot anatomy; instead, they used pre-fabricated wedges, which may have confounded these results.

Seven articles were identified investigating the effect of wedged insoles on BRFs without considering covariates. In a study in which the wedges were customised to individual dynamic barefoot plantar pressure data, all but two subjects reduced peak rearfoot eversion angles compared to footwear without wedges [94]. This finding suggests that wedges bear high potential when individualised to foot pressure mapping. Pre-fabricated medial wedges have proven effective in decreasing maximal rearfoot eversion angles and eversion range of motion [94–97]. When comparing footwear with and without wedges, non-systematic changes in vertical GRF loading rates and knee abduction angular impulse have been reported [95, 96, 98, 94, 99, 100]. When the mediolateral elevation was systematically altered, no perceived comfort and stability changes were reported [95]. Moreover, neither medially nor laterally wedged insoles were able to relieve runners of patellofemoral pain [99]. One study introduced forefoot wedges with systematic changes in elevation; however, no changes in ground contact times were reported [101].

In summary, the response to medially wedged insoles is independent for shorter running durations (< 30 min) but may help runners with a history of PFPS to minimise knee abduction angular impulses; however, the effect for longer running durations (> 30 min) remains unknown. The limited literature shows that joint alignments, injury history, and knee pain are less relevant covariates when individualising wedged insoles. Medially wedged insoles might sufficiently limit rearfoot eversion movement and support runners with a history of AT, TSF, and MTSS to reduce reinjury. To attenuate vertical GRF loading rates, runners with a history of PF might refer to other FDF modifications to reduce the overuse injury risk.

#### Arch support systems

Arch support systems help the foot by storing and releasing elastic energy and preventing arch collapse during high loading [102]. Foot arches can be classified as flat/low, normal, or high [103]. Within the three groups, low-arched runners may exhibit greater eversion movement and velocity than high-arched runners [104]. Arch support systems can be integrated into the midsole or achieved through custom-made insoles shaped into the foot arch [105].

Our review found seven articles, four of which examined the effect of arch support systems on running biomechanics with a covariate (Fig. 4, Supplementary Table 5). Two studies used foot arch height as the covariate, and they found that high-arched runners reduced vertical GRF loading rates in a shoe without arch support, while low-arched runners reduced loading rates in a shoe with arch support. However, both foot arch types experienced reduced rearfoot eversion in a motion control shoe [106]. With a subset of this data, no changes in rearfoot eversion movements for runners with different foot arch types were observed when running in shoes with and without arch support systems during a prolonged run [107]. One article accounted for the runner's foot strike pattern and found that rearfoot strikers decreased ground contact time in footwear without arch support [108]. In contrast, forefoot strikers reduced contact time in a shoe with arch support [108]. The same study found that forefoot strikers in minimal footwear reduced vertical GRF loading rates, but rearfoot strikers did not. Furthermore, training for three months in footwear with a custom-made arch support system reduced rearfoot eversion [105].

We identified three articles investigating the effect of arch support systems on BRFs without considering covariates. A study involving female runners found no effect of arch support on vertical GRF loading rates, peak rearfoot eversion angles, and peak femur rotation angles [46]. Another study also found unchanged rearfoot eversion movements (peak eversion angle and rearfoot inversion at initial ground contact) and knee abduction angles when runners with AT symptoms ran in footwear with and without arch support [109]. Although BRFs were unchanged, a 92% relief of AT symptoms was reported when wearing an insole with custom-made arch support. Finally, one study found unchanged ground contact times when running in midsoles with 20 mm and 24 mm high arch support elevations [101].

The limited literature suggests that arch support systems can potentially reduce BRFs for runners with different arch heights and a history of PF. Runner's foot strike pattern might be considered when individualising arch support systems. When individualising arch support systems to minimise BRFs associated with PFPS (ground contact time) and PF (vertical GRF loading rate), forefoot strikers might benefit from less arch support than rearfoot strikers. Moreover, customised arch support systems enhance comfort perception without changes in peak knee abduction angles and vertical GRF loading rates. Arch support might reduce rearfoot eversion movements and thus have the potential for individualisation for runners with a history of AT, TSF, and MTSS. BRFs related to ITBS (peak femur rotation angle and peak knee abduction angles) seem to change marginally and unsystematically with arch support.

#### Heel flares

Flares can be described as a projection of the midsole and outsole extending beyond the upper [25]. Flares can be placed medially or laterally along the outline of the midsole and were introduced to alter the rearfoot eversion angle, thus increasing foot stability by changing the ankle joint moment arm [110–112].

After examining all articles, we identified five matching our inclusion criteria (Fig. 4, Supplementary Table 6). None of these articles investigated the effect of a covariate.

Concerning BRFs, one study altered the medial heel flare from 0° to 15°, and 30°. The 2D video-based analysis indicated higher rearfoot eversion movement in footwear without heel flares [81]. In the same study, runners running in shoes with the most extreme medial heel flare modification had, on average, lower rearfoot eversion range of motion than in shoes with less or without heel flares. These findings were supported by other research showing that footwear with heel flares can reduce the magnitude of rearfoot eversion across the entire stance phase but does not seem to reduce vertical GRF loading rates [110, 112, 113]. On the contrary, one study with only five runners did not show that rearfoot eversion movement (at initial ground contact, peak, and range of motion) changes when running in footwear with different heel flares [111]. From a perception perspective, heel flares can improve perceived foot stability [112].

None of the articles considered covariates (e.g., foot strike pattern), highlighting future research potential. Although we found diverse results regarding rearfoot eversion movement, midsoles with heel flares might reduce BRFs linked to AT, TSF, or MTSS. Based on the very limited body of literature, midsoles with heel flares are insufficient for reducing vertical GRF loading rates, and individualised heel flares may not target runners with a history of PF.

#### Crash pads

Crash pads are elements incorporated into the posterior-lateral midsole using softer foams, segmented geometries, air pockets, or gel-filled patches. Crash pads in the rearfoot area aim to attenuate the GRF and reduce the GRF's lever arm to the ankle joint [114].

After assessing articles for their eligibility, we identified three articles matching our inclusion criteria (Fig. 4, Supplementary Table 7). Out of the three articles, one study considered the fatigue status of female runners as a covariate. As the runners' fatigue increased, wearing footwear without crash pads increased vertical GRF loading rates compared to the non-fatigue state. However, running in footwear with crash pads maintained consistent vertical GRF loading rates, even as the runners' fatigue increased. [115]. The same study found no effect of fatigue on the peak free moment amplitude.

When not considering covariates, two studies found reduced rearfoot inversion angles at touchdown in footwear with smaller compared to larger crash pad dimensions. However, there were no differences in peak rearfoot eversion angles during the stance phase of running and unsystematic changes in vertical GRF loading rates [114, 116]. Crash pad modifications did not affect the peak free moment amplitude, ground contact time, and rearfoot eversion range of motion [114–116]. Changes in crash pad dimensions do not seem to influence the runner's comfort perception [114]. However, they may provide an essential tool for individualisation to tune midsole cushioning properties without increasing stack height which has been shown to increase rearfoot eversion [81].

Fatigue seems to be a relevant covariate when individualising crash pads to minimise vertical GRF loading rates, thus, might lower the risk of developing PF. However, runners with a history of TSF might need other individualised FDF to lower peak free moment amplitudes. Increasing crash pad height might help runners with plantar fascia complaints by lowering the vertical GRF loading rates. Runners with a history of AT, TSF, or MTSS might benefit from crash pads by reducing rearfoot eversion movement. Surprisingly, although the FDF aimed at attenuating the peak impulse, we have identified only two studies that have analysed vertical GRF loading rate as BRF.

### Other footwear design features

#### Rocker

Rockers in running shoes aim to reduce the strain on the toes, foot, and ankle by altering the midsole's curvature in the anterior–posterior direction, positioning the apex near the metatarsal heads, and enhancing the midstance-to-push-off transition for a smoother heel-to-toe rolling motion [117].

Each of the three identified articles considered a covariate in their analysis (Fig. 4, Supplementary Table 8). One study considered running speeds as a covariate. Although running at higher speeds increases the vertical GRF loading rate, no changes in GRF loading rates were observed between shoes with and without rocker [118]. Two studies considered the foot strike pattern and found that a toe spring starting closer to the midfoot reduced pressure in the forefoot compared to a standard rocker placed at 65% of the shoe length [119]. However, runners perceived the traditional rocker as more comfortable. When compared to shoes without rockers, one study found that a rocker shoe reduced ground contact time but did not affect knee flexion angles at initial ground contact [120].

The number of studies addressing injury-specific BRFs and the effects of rocker designs is limited. Rockers involve different levels of FDF (stack height, cushioning), and therefore it is difficult to assign a specific feature to a specific BRF. More research is needed to understand if certain covariates can cause a specific change in BRFs and how different FDFs that combine a rocker design need to be tuned for individualisation.

#### Outsole profile

A shoe's outsole interacts with the running surface and requires attributes like traction, waterproofness, durability, and puncture resistance [121]. Material robustness might be related to running shoe comfort, and high traction might increase free moment amplitudes associated with TSF [122].

After assessing all articles for eligibility, we could not identify any articles matching our predefined inclusion criteria (Fig. 4). Future studies might use wearable sensors or markerless tracking systems to analyse runners wearing shoes with different outsole profiles on natural surfaces.

#### Flex grooves

Flex grooves and zones are included in outsoles and midsoles to enhance flexibility, facilitating metatarsophalangeal joint movement and shock absorption. Their placement is essential for the joint's variable axis and should be individualised based on foot measurements. Recent 3D measurements indicate significant variation, underscoring the need for personalized flexible zones [123].

Our literature search identified one article matching our predefined inclusion criteria (Fig. 4, Supplementary Table 9). This article considered running speed as a covariate. In this study, the midsole flexibility was altered by cuts with different orientations at the heel region. Although interaction effects were only marginal when jogging or running in footwear with different groove designs, a 10% lower vertical GRF loading rate was observed in the midsole with grooves compared to the midsoles without grooves at the rearfoot [124]. Interestingly, footwear with greater flexibility is perceived as more comfortable than midsoles with less flexibility [125, 126].

While there is limited research on the impact of flex grooves on relevant BRFs for common RRI, one identified article found that they can reduce vertical GRF loading rates, suggesting that flex grooves may be customised for runners with PF.

#### Longitudinal bending stiffness

The longitudinal bending stiffness can impact the running economy by optimising energy return and kinematics of the metatarsal joint and force application [127–131]. The bending stiffness can be modified by adding reinforcement materials or changing the geometry of stiff midsole compounds. The optimal bending stiffness depends on factors such as running speed and body weight [128, 132].

Our literature search identified eleven articles, of which four accounted for a covariate (Fig. 4, Supplementary Table 10). All four articles considered running speed as a covariate. None of these articles found a significant interaction effect on BRFs when running in footwear with different longitudinal bending stiffness at different running speeds [133–136]. Independent of running speed, studies reported reduced ground contact times when running in shoes with lower bending stiffness, while one article found unchanged ground contact times [136].

When not considering covariates, three studies found no changes in the GRF braking impulse when running in shoes with different bending stiffness [135, 137, 138]. On the contrary, a reduction in GRF braking impulse in footwear with higher bending stiffness was found in one study [134]. Eight articles found a reduction in the ground contact time [130, 133–135, 137–139], and two found unchanged ground contact times [134, 140] when running in midsoles with lower bending stiffness. Although studies found lower vertical GRF loading rates [140] and increased comfort perception [135] when athletes ran in more flexible than stiffer midsoles, the relationship between BRFs and injury development when altering the longitudinal bending stiffness has not been sufficiently studied yet, but first studies have evolved showing that bones stress injuries might increase when switching to footwear with carbon fibre plates [18].

The limited body of literature suggests that fitting longitudinal bending stiffness to the runner's needs may help with treating PFPS. While reduced bending stiffness can reduce ground contact time, higher stiffness can reduce ground reaction force braking impulse. However, injury prevention and reinjury risk minimisation under the light of different longitudinal bending stiffness has been insufficiently investigated. Furthermore, flexible midsoles with lower longitudinal bending stiffness might reduce vertical GRF loading rates and potentially help runners with a history of PF.

### The upper

The running shoe upper is comprised of a textile fabric and lacing system that couple the foot and shoe, with reinforcement materials used for stability and breathability. An optimal fit depends on individual foot morphology, while insufficient coupling can negate benefits from other design features. Moreover, excessive pressure can affect comfort by restricting blood supply, making individualisation important [141]. Since foot dimensions differ across sexes, ages, and ethnic origins, individualised upper bears great potential for individualisation [142].

#### Upper fabric

Our systematic literature search identified two articles investigating the effect of different upper modifications (Fig. 4, Supplementary Table 11). None of the articles considered covariates [53, 143].

The data indicates that a soft-sewed structured fabric reduces knee abduction angles and vertical GRF loading rates compared to a minimalist heat fusion fabric. Furthermore, the ground contact time was reduced when running in minimalist heat fusion fabric.

The current body of literature is insufficient to give recommendations for upper individualisation concerning the reduction of BRFs. Based on the limited results, upper materials might be individualised to the runner's preference.

#### Lacing

Five articles have investigated the effect of lacing on the lower extremity joint biomechanics or subjective comfort perception (Fig. 4, Supplementary Table 12).

One of five studies considered the runner's experience as a covariate. The researchers found that low-level runners perceived an irregularly (skipping eyelets) laced running shoe as more stable and comfortable than high-level runners who preferred a regular high and tight lacing pattern [144].

We identified four studies analysing BRFs without accounting for covariates. According to a study, running shoes with traditional lacing and elastic upper material were perceived as more comfortable than footwear without lacing [145]. When running in shoes with various lacings, two studies found no significant difference in the rearfoot eversion angle at initial contact [145, 146]. The same studies found a reduction in the peak rearfoot eversion angle when running in traditionally laced shoes compared to those without traditional lacing. However, another study systematically changed lacing patterns and could not find any differences in the peak rearfoot eversion angle [147]. Different types of lacing patterns, particularly high- and tightly-laced shoes, have been shown to reduce vertical GRF loading rate at the cost of comfort [144, 148].

Studies analysing BRFs and considering relevant covariates, e.g., foot shape, are required in the future. Notably, no studies have measured the foot-shoe coupling or the relative movement of the foot within the shoe, highlighting the potential for future research to determine individualised fits and their interactions with other FDF. Since peak rearfoot eversion angles and vertical GRF loading rates are reported to be lower when running in tightly and high-laced shoes, runners with a history of MTSS and TSF might target individualised lacing systems.

## Discussion and future perspectives

Our findings suggest that studies assessing the effects of footwear on BRFs rarely take covariates into account. The literature considering covariates and BRFs is limited for heel flares, midsole longitudinal flexibility/stiffness, and upper, and rocker modifications. Especially the latter seems to have a high potential for individualised footwear solutions for rear- and forefoot strikers since remarkably different responses to non-BRFs have been reported [120].

The response of BRFs associated with MTSS, TSF, and AT to different FDF has been studied in greater detail. The recently published systematic review identified rearfoot eversion movements (peak and range of motion) as BRFs [10]. However, only considering eversion movements as BRFs might overlook the complexity of the tri-planar motion of the foot and ankle complex. Although injuries around the knee joint are the most common [5], little research has been dedicated to understanding how running footwear modifications can redistribute knee joint stress.

Currently, there are some indicators about which BRFs can be modified via certain FDF. The effect of different types of footwear on covariates such as environmental constraints, athlete anthropometry, and level of experience and their influence on BRFs has been reported (albeit limited). However, future research should investigate the effect of shoe type on other important covariates, such as training load, fatigue, running distance, and step count linked to BRFs. Advantages in markerless tracking systems and wearable sensors might leverage big data collections to analyse runners and account for relevant covariates in real-world scenarios. Moreover, the use of classical repeated-measures statistics might blur the understanding of a systematic footwear response. Although repeated-measures statistics help determine an "average" response to (two or more) footwear conditions, it does not help to understand whether these responses are systematic. Using classical mean comparisons and neglecting the individual response overlooks the complexity of footwear and shoe interaction. Reporting additional statistical metrics, e.g., individual data or rank correlations, might help other researchers understand whether a footwear response was systematic or individual.

The findings of this literature review need to be interpreted in light of some limitations. First, we included all articles that analysed BRFs or considered a covariate in their analysis. The BRFs identified by the systematic review show high uncertainty, and there are contradictory results across studies [149]. Although the causality of some BRFs remains questionable, precise measurement of BRFs is one of the few injury risk estimators footwear manufacturers, coaches, and athletes can rely on at the moment. Second, the interaction of FDF cannot be excluded (Supplementary Table 1–12). A large number of the included studies varied multiple FDF within the same experiment without controlling for the interaction effect of the modification. For example, when comparing a minimal to a neutral and motion control shoe, it is unclear which FDF have been modified and affect the biomechanical outcome. Therefore, more studies addressing FDF systematically and considering FDF interactions should be performed in the future.

## Conclusion

There is high uncertainty on relevant covariates affecting the biomechanical response to different FDF. The aetiology behind RRI development is multifactorial, and determining a runner's risk profile is anything but trivial [150]. In running, inappropriate footwear does not per se cause an RRI. Instead, it affects the individual injury risk profile through interaction with other factors, e.g., biomechanics, training load and anthropometrics (Fig. 1). However, individual structure-specific stress capacities as covariates need to be considered when establishing a cause-effect relationship between footwear and injury.

In conclusion, an FDF may increase the magnitude of a BRF for one injury and decrease that of another BRF for another injury, which suggests that footwear needs to be individualised and injury-specific. However, more research is required to identify relevant covariates and consider training load characteristics. In particular, the runner's injury risk profile should be assessed in real-world scenarios to better understand the response to footwear modifications. Because little is known about important covariates, creating an individualised shoe may still require extensive laboratory-based running tests to determine BRFs and modify them with FDF. Although we reported that certain FDF bear the potential for individualisation and for reducing injury-specific BRFs, it is not recommended to individualise multiple FDF at once. Instead, FDF should be individualised gradually to avoid interactions of FDF or over-designing and simultaneously maximise footwear comfort.

### Supplementary Information

**Additional file 1. **Preferred Reporting Items for Systematic reviews and Meta-Analyses extension for Scoping Reviews (PRISMA-ScR) Checklist.

**Additional file 2: Supplementary Materials Appendix 2.** Search strings.

**Additional file 3: Table 1.** Summary of the study characteristics, publication details, population details, data collection protocol, covariates, and biomechanical outcome variables for midsole hardness and cushioning modifications with a detailed description of the footwear studied. **Table 2.** Summary of the study characteristics, publication details, population details, data collection protocol, covariates, and biomechanical outcome variables for midsole geometrical modifications (heel-toe drop and midsole thickness) with a detailed description of the footwear studied. **Table 3.** Summary of the study characteristics, publication details, population details, data collection protocol, covariates, and biomechanical outcome variables for postings with a detailed description of the footwear studied. **Table 4.** Summary of the study characteristics, publication details, population details, data collection protocol, covariates, and biomechanical outcome variables for wedges with a detailed description of the footwear studied. **Table 5.** Summary of the study characteristics, publication details, population details, data collection protocol, covariates, and biomechanical outcome variables for arch support systems with a detailed description of the footwear studied. **Table 6.** Summary of the study characteristics, publication details, population details, data collection protocol, covariates, and biomechanical outcome variables for heel flare modifications with a detailed description of the footwear studied. **Table 7.** Summary of the study characteristics, publication details, population details, data collection protocol, covariates, and biomechanical outcome variables for crash pad with a detailed description of the footwear studied. **Table 8.** Summary of the study characteristics, publication details, population details, data collection protocol, covariates, and biomechanical outcome variables for rocker modifications with a detailed description of the footwear studied. **Table 9.** Summary of the study characteristics, publication details, population details, data collection protocol, covariates, and biomechanical outcome variables for flex grooves modificaions with a detailed description of the footwear studied. **Table 10.** Summary of the study characteristics, publication details, population details, data collection protocol, covariates, and biomechanical outcome variables for longitudinal bending stiffness and flexibility modificaions with a detailed description of the footwear studied. **Table 11.** Summary of the study characteristics, publication details, population details, data collection protocol, covariates, and biomechanical outcome variables for upper modificaions with a detailed description of the footwear studied. **Table 12.** Summary of the study characteristics, publication details, population details, data collection protocol, covariates, and biomechanical outcome variables for lacing modificaions with a detailed description of the footwear studied.

**Additional file 4: Table 1S.** Running-related biomechanical risk factors and their level of evidence for different types of overuse injuries identified by Willwacher et al. [15]. Arrows pointing upwards indicate that an elevation of the biomechanical risk factor is associated with a specific overuse injury, arrows pointing downwards interpreted vice versa. Bolded biomechanical risk factors were identified in prospective studies; at least two independent retrospective studies identified unbolded biomechanical risk factors. A detailed description of the biomechanical risk factors is provided below the table.

## Data Availability

The results of the electronic search are available in the supplementary information.

## Abbreviations

AT: Achilles tendinopathy
BRF(s): Biomechanical risk factor(s)
FDF: Footwear design feature
GRF: Ground reaction force
ITBS: Iliotibial band syndrome
MTSS: Medial tibial stress syndrome
PF: Plantar fasciitis
PFPS: Patellofemoral pain syndrome
RRI: Running-related overuse injury
TSF: Tibial stress fractures
