# Supplementary material for: Towards functionally individualised designed footwear recommendation for overuse injury prevention: a scoping review
Source: BMC Sports Sci Med Rehabil. 2023 Nov 11;15:152. doi: 10.1186/s13102-023-00760-x (PMC10638717; doi:10.1186/s13102-023-00760-x)
Supplement: Supplementary file 2 — Additional file 2: Supplementary Materials Appendix 2. Search strings. [file 13102_2023_760_MOESM2_ESM.docx]

**Supplementary Materials Appendix 2 – Search strings**

**Towards functionally individualized designed footwear recommendation for overuse injury prevention: A Scoping Review**

Patrick Mai^1,4^, Leon Robertz^1^, Johanna Robbin^4^, Kevin Bill^1^, Gillian Weir^2^, Markus Kurz^5^, Matthieu B. Trudeau^3^, Karsten Hollander^6^, Joseph Hamill^2^, and Steffen Willwacher^1,4^

^1^ Institute of Biomechanics and Orthopaedics, German Sport University Cologne, Cologne, Germany

^2^ Biomechanics Laboratory, University of Massachusetts Amherst, Amherst, MA, USA

^3^ Google, United States

^4^ Department of Mechanical and Process Engineering, Offenburg University, Offenburg, Germany

^5^ Sports Tech Research Centre, Mid Sweden University, Östersund, Sweden

^6^ Institute of Interdisciplinary Exercise and Sports Medicine, Medical School Hamburg, Hamburg, Germany

Table 1S: Search string for each footwear design feature used for the PubMed database.

| Footwear Design Feature | |  |  |  |  |  | |  |
| --- | --- | --- | --- | --- | --- | --- | --- | --- |
| Midsole hardness & cushioning | (runn* OR jogg*) | | AND | (hardness OR cushion*) | AND | | (footwear OR shoe OR shod) | |
| Heel-toe-drop: |  |  |  | (heel-toe-drop OR drop OR stack) |  |  |  |  |
| Midsole thickness: |  |  |  | (thick*) |  |  |  |  |
| Postings: |  |  |  | (posting* OR posted) |  |  |  |  |
| Wedges: |  |  |  | (wedge*) |  |  |  |  |
| Arch support systems: |  |  |  | (arch AND support*) |  |  |  |  |
| Heel flares: |  |  |  | (heel AND flar*) |  |  |  |  |
| Crash pads: |  |  |  | (crash AND pad*) |  |  |  |  |
| Rocker: |  |  |  | (rock* OR (toe* AND spring*)) |  |  |  |  |
| Flex grooves: |  |  |  | (groov*) |  |  |  |  |
| Longitudinal bending stiffness: |  |  |  | (long* AND bending AND stiffness) |  |  |  |  |
| Upper: |  |  |  | (upper* OR vamp*) |  |  |  |  |
| Lacing: |  |  |  | (lac OR lacing) |  |  |  |  |
| Outsole profile & traction: |  |  |  | (outsole OR profile OR traction) |  |  |  |  |
